# PPAR-δ as a prognostic biomarker and its association with immune infiltrates in breast cancer PPAR-δ as a prognostic biomarker and its association with immune infiltrates in breast cancer

**DOI:** 10.7150/jca.81430

**Published:** 2023-04-17

**Authors:** Zonghan Wang, Hao Dong, Wei Li, Fujun Han, Lei Zhao

**Affiliations:** 1 Department of the Oncology, Cancer Center, The First Hospital of Jilin University, Changchun, Jilin, China; 2 Key Laboratory of Organ Regeneration & Transplantation of the Ministry of Education, The First Hospital of Jilin University, Changchun, Jilin, China; 3 National-Local Joint Engineering Laboratory of Animal Models for Human Diseases, Jilin University, Changchun, Jilin, China; 4 Department of the Gastrointestinal Nutrition and Hernia Surgery, The Second Hospital of Jilin University, Changchun, Jilin, China; 5 Department of Colorectal and Anal Surgery, The Second Hospital of Jilin University, Changchun, Jilin, China

**Keywords:** PPAR-δ, biomarker, triple-negative breast cancer, immune infiltration, prognosis

## Abstract

While peroxisome proliferator-activated receptor δ (PPAR-δ) and its associated signaling pathways have been shown to play an important regulatory role in various malignant tumors, in breast cancer, its potential influence on immune infiltration and its ability to serve as a prognostic marker remains unclear. BRCA patient samples with matched paracancerous samples were obtained from The Cancer Genome Atlas (TCGA). PPAR-δ expression, its potential effect on immune cell infiltration and its association to clinicopathological features were examined. Gene Ontology (GO) analysis, Kyoto Encyclopedia of Genes and Genomes (KEGG), Gene Set Enrichment Analysis (GSEA) and Single-Sample Gene Set Enrichment Analysis (ssGSEA) were utilized for functional and pathway enrichment and to quantify the extent of immune cell infiltration. Kaplan-Meier analysis and Cox regression analysis (nomogram) were performed to assess the association between PPAR- δ and predicted survival. To confirm these findings, an allograft tumor mouse model was generated and treated with a PPAR-δ inhibitor to examine the role of PPAR-δ expression *in vivo*; while immunohistochemistry (IHC) was performed to examine PPAR-δ expression in paired BRCA patient samples *in vitro*. Overall, the findings presented herein suggest that PPAR-δ plays a crucial role in breast cancer progression and prognosis and may serve as a survival predictive biomarker.

## Introduction

In a 2022 report issued by *CA: A cancer Journal for Clinicians*, 2.26 million breast cancer cases were reported worldwide, with numbers surpassing lung cancer for the first time and it gaining the title of "first cancer" in the world. Furthermore, breast cancer has become the most common malignancy in women and one of the leading causes of cancer-related deaths in women, with 680,000 deaths reported in 2021 1-3. Moreover, young women (age < 40) diagnosed with breast cancer have a higher predisposition towards a poor clinical prognosis and early-stage metastasis. In recent years, immunotherapy, specifically immune checkpoint modulation (immune checkpoint blockade), has provided a promising treatment for lung cancer, skin cancer, bowel cancer and certain breast cancers 4, 5. In 2019, the U.S. Food and Drug Administration (FDA) approved the use of the immune checkpoint inhibitor atezolizumab, an anti-PD-L1 monoclonal antibody, in the treatment of metastatic triple-negative breast cancer patients; with the combination of atezolizumab and nab-paclitaxel significantly prolonging the survival time in these breast cancer patients 6. However, since anti-PD-L1 monoclonal antibodies have only been shown to benefit triple-negative breast cancer patients (~18%), other tumor therapeutic targets are also being explored to improve patient prognoses 7.

Peroxisome proliferator-activated receptors (PPARs) are members of a ligand-activated nuclear transcription factor superfamily that includes three isotypes, PPARα, PPARβ/δ and PPARγ, with PPAR-δ being the less studied. PPAR-δ plays an important regulatory role in inflammation, atherosclerosis, insulin resistance and the regulation of glucose metabolism, tumor and obesity. Furthermore, recent studies have suggested possible new clinical applications for PPAR agonists and antagonists in cancer treatment 8, 9. In a colitis-associated colon cancer mouse model, PPARβ/δ overexpression promoted tumorigenesis in mice 10 and increased IL-6 expression and STAT3 phosphorylation, while concomitant 15-Lipoxygenase-1 expression suppressed these effects 11. Furthermore, increased PPARβ/δ expression was detected in human melanoma compared with normal skin 12. Moreover, PPARβ/δ activation using GW0742 or GW501516 has been shown to inhibit proliferation in different melanoma cell lines 12, 13 due to direct transcriptional repression of Wilms' tumor suppressor (WT1) and its downstream target genes zyxin and nestin 14-16.

In this study, RNA-sequencing data, BRCA and various other cancers, was retrieved from the TCGA database and bioinformatics and statistical analyses, including differentially expressed gene (DEG) identification, Gene Ontology (GO), Kyoto Encyclopedia of Genes and Genomes (KEGG), Gene Set Enrichment Analysis (GSEA) and Single-Sample Gene Set Enrichment Analysis (ssGSEA), were employed. To determine the survival predictive ability of PPAR-δ, a nomogram was constructed. Finally, PPAR-δ levels were evaluated in matched BRCA patient samples and the inhibition of PPAR-δ was evaluated using an allograft tumor mouse model.

## Material and Methods

### Data source and preprocessing

Gene expression data, with associated clinical information, from BRCA projects (included 113 normal and 1109 tumor tissues, workflow type: HTSeq-FPKM) were collected from the Cancer Genome Atlas (TCGA) database, with duplicate samples removed. Next, level 3 HTSeq-FPKM data were transformed into TPM (transcripts per million reads), and the TPM data of 1109 BRCA patients were used for further analyses (Supplementary Table 1). Unavailable or unknown clinical features were regarded as missing values.

### PPAR-δ differential expression in BRCA samples

The filtered data obtained from the TCGA database was analyzed via scatter plots, with the disease state (tumor or normal) as the variable and differential PPAR-δ expression examined. BRCA samples with PPAR-δ expression above or below the median value were defined as PPAR-δ-high or PPAR-δ-low, respectively. Analysis of differential genes (DEGs) between PPAR-δ-high and -low expression BRCA groups.

DEGs between PPAR-δ-high and PPAR-δ-low samples obtained from the TCGA datasets were identified by performing an unpaired Student's t-test, within the DESeq2 (3.8) package 17. Genes were deemed significantly differentially expressed if an adjusted P value < 0.05 and an absolute FC > 1.5 were obtained (Supplementary Table 2). All identified DEGs were then analyzed using volcano plots.

### Functional enrichment and analysis of immune cell infiltration

The identified DEGs were further analyzed using Metascape (http://metascape.org), with P < 0.01 and an enrichment factor > 1.5 required to be deemed statistically significant 18. Gene Set Enrichment Analysis (GSEA) was employed to determine associations between the PPAR-δ-high and -low groups and associated signal pathways. To confirm differential pathways, a permutation test, with 1,000 permutations performed, was utilized, and genes were deemed significantly associated at P < 0.01 and FDR < 0.25. Statistical analysis and plot construction were conducted using clusterProfiler (3.8.0) within the R package 19. Relative immune cell infiltration levels were quantified using single-sample GSEA (ssGSEA, version 1.40.1) based on published signature gene lists 20, with a diverse set of adaptive and innate immune cell types examined (Supplementary Table 4). To explore the correlation between PPAR-δ expression levels and immune cell infiltration levels, a Wilcoxon rank-sum test and Spearman correlation analysis were employed.

### Clinical statistical analysis on prognosis, model construction and evaluation

All statistical analyses were performed utilizing the R package (v3.6.2). Potential correlations between clinical pathologic features and PPAR-δ expression for the TCGA dataset were analyzed with a Wilcoxon signed-rank sum test and logistic regression. Clinicopathological characteristics associated with the overall survival (OS), progression-free interval (PFI), and disease-specific survival (DSS) were examined using Cox regression analysis and the Kaplan-Meier method, with multivariate Cox analysis used to evaluate the influence of PPAR-δ expression on survival and other clinical characteristics. PPAR-δ expression cut-off values were determined based on the median value, with P < 0.05 deemed significant in all tests. Based on the Cox regression models, the independent prognostic factors obtained from multivariate analysis were used to establish nomograms that enable the individualization of the predicted survival probabilities for 3, 5, or 10 years. The RMS package (version: 5.1-4; https://cran.rproject.org/web/packages/rms/index.html) was employed to generate nomograms that included significant clinical characteristics and calibration plots. The calibration curves were graphically assessed by mapping the nomogram-predicted probabilities against the observed occurrences, with a 45° line representing the ideal predictive values. A concordance index (C-index) was used to determine the discrimination of the nomogram, and it was calculated using a bootstrap approach with 1,000 resamples. The predictive accuracies of the nomogram and separate prognostic factors were compared using the C-index. All statistical tests were two-tailed, with P < 0.05 deemed significant.

### Breast cancer cell line

The 4T1 cells (ATCC) were incubated with RPMI-1640 medium (Gibco, Carlsbad, CA) containing 10% fetal bovine serum in an incubator at 37 °C and 5% CO_2_. The PPAR-δ in 4T1 cells was antagonized using different concentrations (1, 5, 10, 20, 40 or 80 µM) of the PPAR-δ antagonist GSK3787 (S8025, Selleck). Total protein extracts were obtained using a total protein extraction kit (KeyGEN Biotech, Jiangsu, China) according to the manufacturer's instructions. Protein concentrations were measured using a BCA protein assay (23227, Thermo Fisher Scientific) according to the manufacturers' protocols to ensure equal protein loading for each sample.

### Western blotting

Protein lysates (30-40 µg) were separated via 12% sodium dodecyl sulfate-polyacrylamide gel electrophoresis (SDS-PAGE). Samples were then transferred to PVDF membranes (Millipore, Billerica, MA, USA). The PVDF membranes were blocked with 5% non-fat milk dissolved in TBST (TBS and 0.01% Tween-20) for 2 h at room temperature, followed by an overnight incubation at 4 °C with p-JAK (#3331, CST, Boston, USA; 1:1000), JAK (#3344, CST, Boston, USA; 1:1000) or Vinculin (4650S, CST, Boston, USA; 1:1000). Finally, the transfer membranes were incubated with horseradish peroxidase-labeled anti-rabbit or anti-goat IgG secondary antibodies (Abcam, Cambridge, UK; 1:5000) for 1 h. Bands were visualized using an enhanced chemiluminescence (ECL) detection kit (KGP1127-1128, KeyGEN Biotech, Jiangsu, China) and imaged using a bio-imaging system. The images were analyzed using the ImageJ software and all samples were examined in triplicate.

### Immunohistochemistry

Paired BRCA breast tissue samples (n =10) were obtained from patients who underwent a mastectomy at the Department of Surgery, The Third Hospital of Jilin University, Changchun, China between January 2019 and December 2021; with the non-malignant paired samples obtained from at least 5 cm away from the tumor. Per institutional guidelines, an ethical review was not required for the human samples that were obtained for this study. All patients signed a written informed consent prior to participating in this study. Tumor tissues were examined via IHC using formalin-fixed paraffin embedded primary tumor tissue. Heat-induced antigen retrieval was performed using 0.1 M citrate buffer (pH 6.0) and autoclaved for 20 min. Endogenous peroxidases were eliminated using 3% hydrogen peroxide and slides were incubated with PPAR-δ antibody (ab137724, Abcam, Cambridge, UK; 1:100) in PBS with 1% BSA overnight at 4 °C. For visualization, slides were incubated with HRP rabbit polymer (Dako REAL EnVision, Glostrup, Denmark) and liquid diaminobenzidine tetrahydrochloride (DAB) plus substrate (Dako). All slides were counterstained with hematoxylin. The IHC results were observed under an Olympus X51 microscope at 400× magnification and were photographed using DP controller software. At least five fields of view were randomly selected from each tissue section, with the integrated optical density of positive cells analyzed using Image pro plus 6.0 software. The relative PPAR-δ protein expression was presented as a mean optical density (MOD), with the MOD = cumulative optical density/positively stained area.

### Animal Models

Female BALB/c mice (6-7 weeks old) were purchased from Charles River Laboratories (Beijing, China) and raised in a pathogen-free environment with free access to food and water. All procedures were approved by the Jilin University Animal Care and Use Committee and were in compliance with the guidelines outlined in the Guide for the Care and Use of Laboratory Animals. To evaluate the role of PPAR-δ *in vivo*, we conducted an *in situ* breast injection with 4T1 cells in healthy 8-week old female BALB/c mice to generate a tumor allograft. After constructing a breast cancer model, the subjects were randomly divided into two equal groups (n = 5), a control group and a PPAR-δ inhibitor group. PPAR-δ was antagonized with GSK3787 inhibitor (10 mg/kg; S8025, Selleck) dissolved in DMSO in preparation for a clear stock solution, which was then dissolved in co-solvent PEG300, Tween-80 and 0.9% normal saline. The final GSK3787 solution contained 10% DMSO, 40% PEG300, 5% Tween-80 and 45% saline. When the tumor size reached 100 mm^3^, the inhibitor was intraperitoneally administered, with treatment given every three days for a total of four doses. Tumors were then harvested for further examination.

### Flow cytometry

Allograft tumor tissue samples were digested into single cell suspensions using tumor dissociation kits (130-096-730, Miltenyi Biotech, Bergisch Gladbach, Germany) and stained with anti-CD45 (103126, Biolegend, San Diego, CA), anti-CD3 (100320, Biolegend, San Diego, CA), anti-CD4 (100550, Biolegend, San Diego, CA), anti-CD11b (557657, BD, Biosciences, San Jose, CA), anti-CD8 (100710, Biolegend, San Diego, CA), anti-CD19 (115554, Biolegend, San Diego, CA) and anti-CD25 (102051, Biolegend, San Diego, CA). Dead cells were excluded using a LIVE/DEAD Fixable Aqua Dead Cell Stain Kit (L34966, Life Technologies, Carlsbad, CA) according to the manufacturer's protocols. Flow cytometry was performed using a Cytek flow cytometer (Cytek Aurora; Cytek Biosciences, Inc., Fremont, CA) and analyzed using FlowJo software (v.10.8.1; BD Biosciences, San Jose, CA).

### Statistical analysis

Statistical analysis was performed with SPSS software version 11.0. Data are expressed as mean ± SD. Differences between two groups were evaluated by Student's t-test. One-way ANOVA was used when comparing multiple groups. P < 0.05 was considered statistically significant. Clinical data analysis of survival and relevant correlations were performed with GraphPad Prism.

## Results

### Up-regulation of PPAR-δ in BRCA

**Figure 1:**
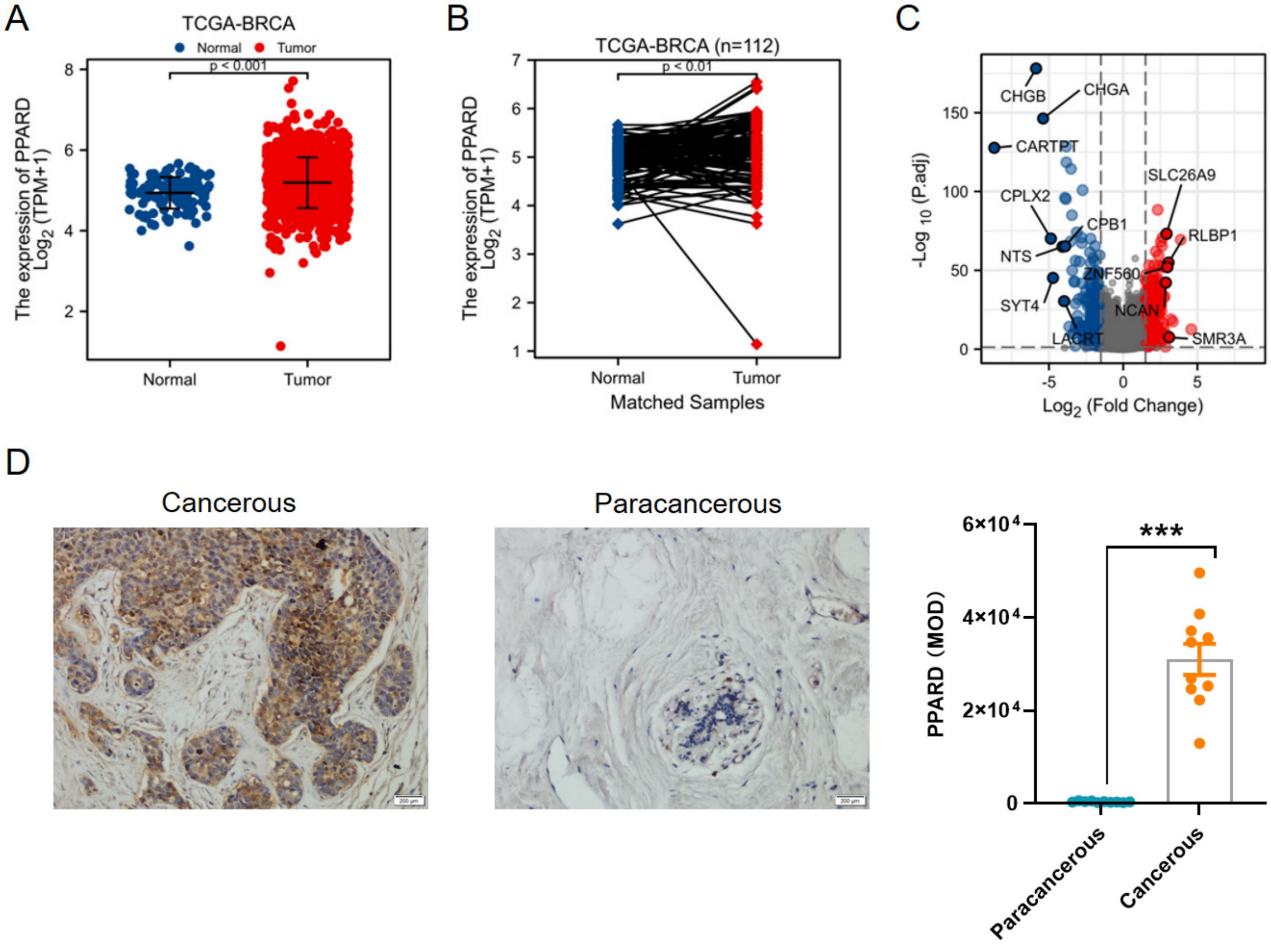
Differential expression of PPAR-δ in BRCA and PPAR-δ related DEGs base on TCGA dataset. (**A**-**B**) Differential PPAR-δ expression in BRCA samples relative to normal samples. (**C**) Volcano plots displaying the most significantly up- or down-regulated DEGs. (**D**) Representative IHC image of differential PPAR-δ expression in obtained patient and matched paracancerous tissue (n = 10 each).

First, PPAR-δ expression was compared between 112 paracancerous samples and 1109 breast infiltrating carcinoma (BRCA) samples obtained from the TCGA dataset, and also showed a significant up-regulation of PPAR-δ in the BRCA samples relative to the paracancerous samples (Figure 1A); with this difference preserved when comparing the 112 matched samples (Figure 1B). To further validate this finding, PPAR-δ expression was examined in 10 BRCA and paracancerous patient samples using IHC (P <0.001; Figure 1D). The obtained BRCA and control samples (TCGA dataset) were then further analyzed, with a total of 597 DEGs, 274 up-regulated and 323 down-regulated, found to be significantly differentially expressed in BRCA relative to the normal samples (adjusted P < 0.05, |Log2-fold change| > 2; Figure 1C and Supplementary Table 2). The identified DEGs in HTSeq-Counts were further analyzed using the DESeq2 package, with relative expression values determined.

### Functional enrichment and analyses of PPAR-δ related genes in BRCA

**Figure 2:**
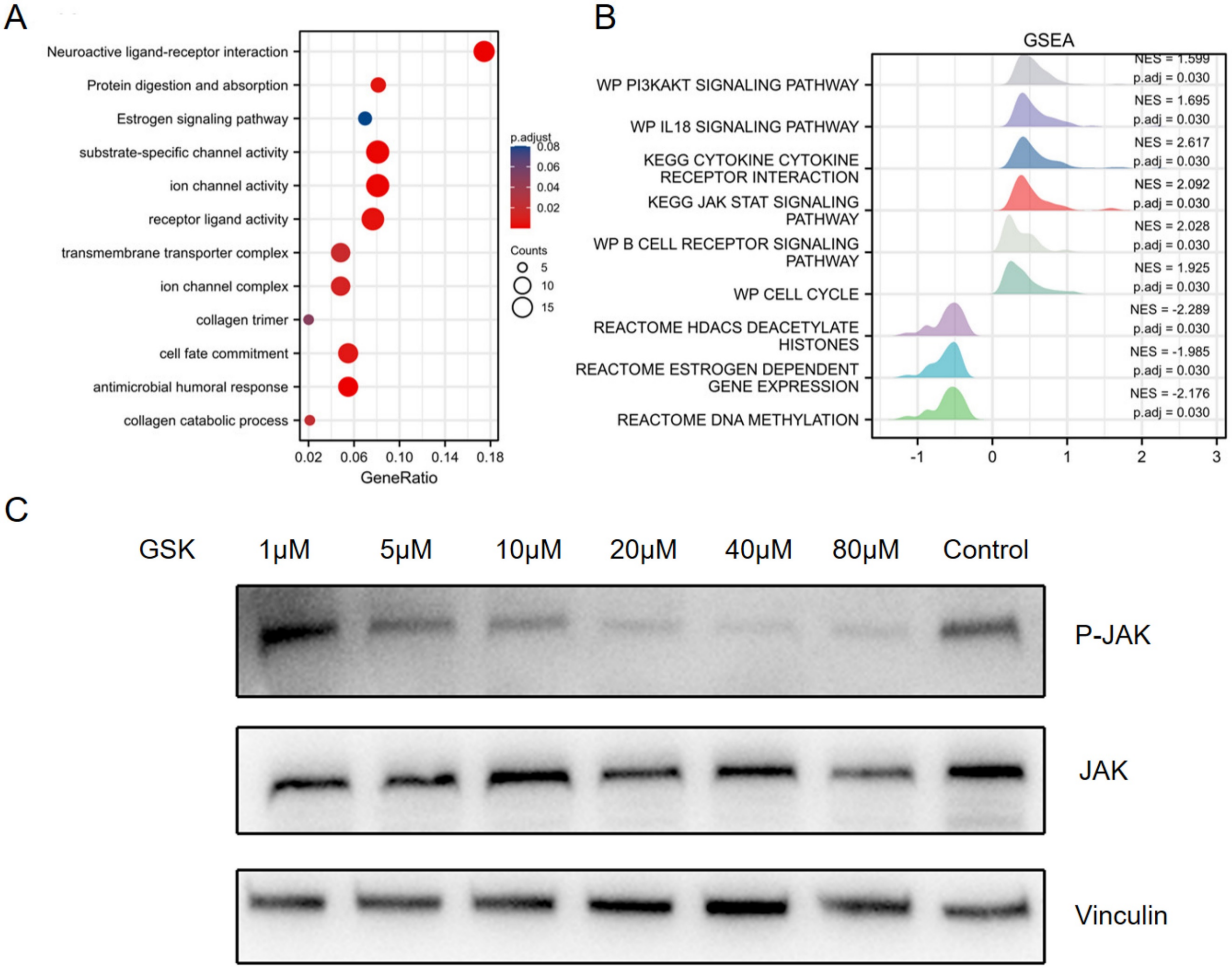
GO and KEGG enrichment analysis of PPAR-δ related DEGs in BRCA based on the TCGA dataset. (**A**) Top 12 enriched genes within the GO biological process function group. (**B**) Pathway enrichment of using gene set enrichment analysis (GSEA). Several pathways and biological processes were differentially enriched in PPAR-δ related BRCA. (**C**) After generating an allograft using 4T1 cells, PPAR-δ was antagonized using GSK3787. Western blot analysis showing that JAK/STAT and p-JAK are inhibited in a dose-dependent manner following GSK3787 treatment. NES, normalized enrichment score; p.adj, adjusted P value.

To further evaluate the identified DEGs and potential PPAR-δ interactions, functional enrichment was performed using Metascape, to include GO and KEGG enrichment. The analysis identified PPAR-δ-related genes involved in the categories of biological processes (BPs), cellular compositions (CCs) and molecular functions (MFs), while KEGG enrich pathways included neuroactive ligand-receptor interaction, protein digestion and absorption and estrogen signaling pathway. Moreover, antimicrobial humoral response, collagen catabolic process and transmembrane transporter complex were also implicated in association with PPAR-δ (Figure 2A and Supplementary Table 3). To identify PPAR-δ related signaling pathways in BRCA, GSEA was performed to further elucidate differences between the PPAR-δ-high and -low groups using a MSigDB collection (c2.cp.v7.2.symbols.gmt [Curated]), with a false discovery rate (FDR) < 0.25 and adjusted P <0.05 required for significance. The most significantly enriched signaling pathways based on their normalized enrichment score (HES) were selected. The identified differentially enriched pathways for the PPAR-δ-high group included HDACS deacetylate histones, estrogen dependent gene expression and DNA methylation; while for the PPAR-δ-low group, pathways included PI3K/AKT, IL-18, JAK/STAT and B cell receptor and cell cycle (Figure 2B, Supplementary Figure 1 and Supplementary Table 3). To further examine these findings, an *in-vitro* cell experiment was constructed, with cells treated with a specific PPAR-δ antagonist (GSK3787) at different concentrations (1, 5, 10, 20, 40 or 80 µM). The results showed that JAK/STAT signaling was inhibited and that JAK phosphorylation was significantly down-regulated in a dose-dependent manner (Figure 2C).

### Correlation between PPAR-δ expression and immune infiltration

**Figure 3:**
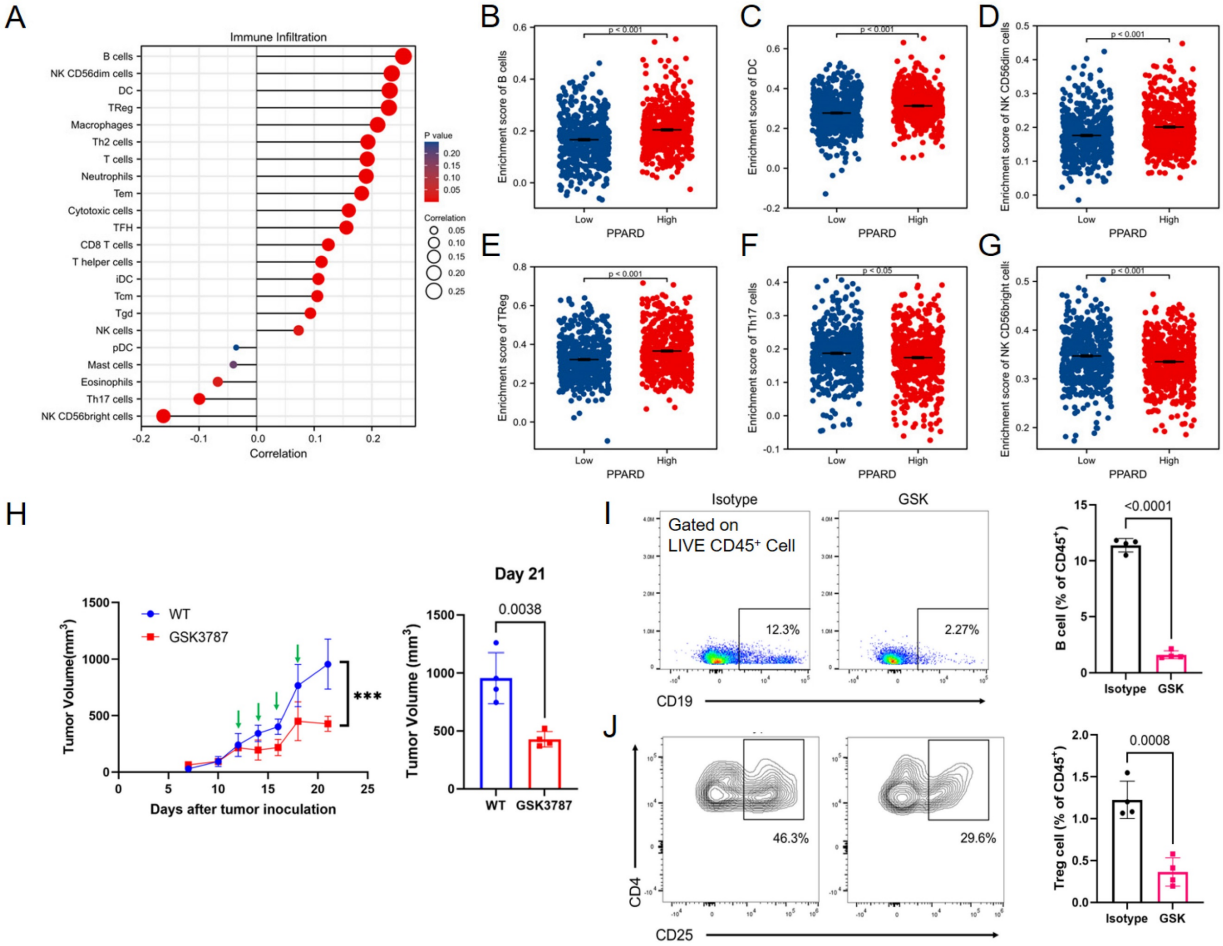
PPAR-δ expression is associated with immune infiltration within the tumor microenvironment. (**A**) Correlations between the relative abundances of 24 immune cells and PPAR-δ expression levels. (**B-G**) Scatter plots showing difference in B cells, DC, macrophages, Treg, NK and Th17 cell infiltration levels between the PPAR-δ-high and -low groups. (**H**) After antagonizing PPAR-δ with GSK3787 in the mouse allograft, tumor growth was inhibited. Green arrows represent dosing time points. (**I, J**) Expressional changes in immune cells within the mouse tumor allografts as analyzed by flow cytometry.

A potential correlation between PPAR-δ expression and immune cell infiltration levels was quantified using ssGSEA and analyzed via spearman correlation (Supplementary Figure 2). The results showed that CD56bright NK cells and helper T17 (Th17) cells were negatively correlated with PPAR-δ expression, and more types of immune cells were positively correlated with the expression of PPAR-δ, including B cells, CD56dim NK cells, dendritic cells, T reg cells, macrophages, helper T2 (Th2) cells, and centriocytes, etc. (Figures 3A-F). Notably, an increase in the number of CD56bright NK cells was noted when PPAR-δ expression was reduced (Figure 3G). CD56^bright^ NK cells express low levels of CD16A and can produce large amounts of IFN-γ and other factors under the stimulation of cytokines to elicit an anti-tumor effect. These findings suggested that inhibiting PPAR-δ expression can enhance the anti-tumor immune response of innate immune cells.

To further evaluate these findings, the allograft tumor mouse model was employed, with the 4T1 tumors being examined via flow cytometry following PPAR-δ antagonizing (GSK3787). The results showed that a proportion of the B cells and Treg cells decreased after inhibition (Figures 3I, J). The results correspond to the previous correlation analysis, and a preliminary guess can be made that the increased expression of PPAR-δ could inhibit the anti-tumor associated immune cell infiltration (CD56bright NK cells) and promote some immune cell species that are not conducive to anti-tumor (Treg cells, etc.). Furthermore, treatment with the PPAR-δ inhibitor significantly stifled tumor growth (Figure 3H).

### Association with PPAR-δ expression and clinicopathological variables

**Figure 4:**
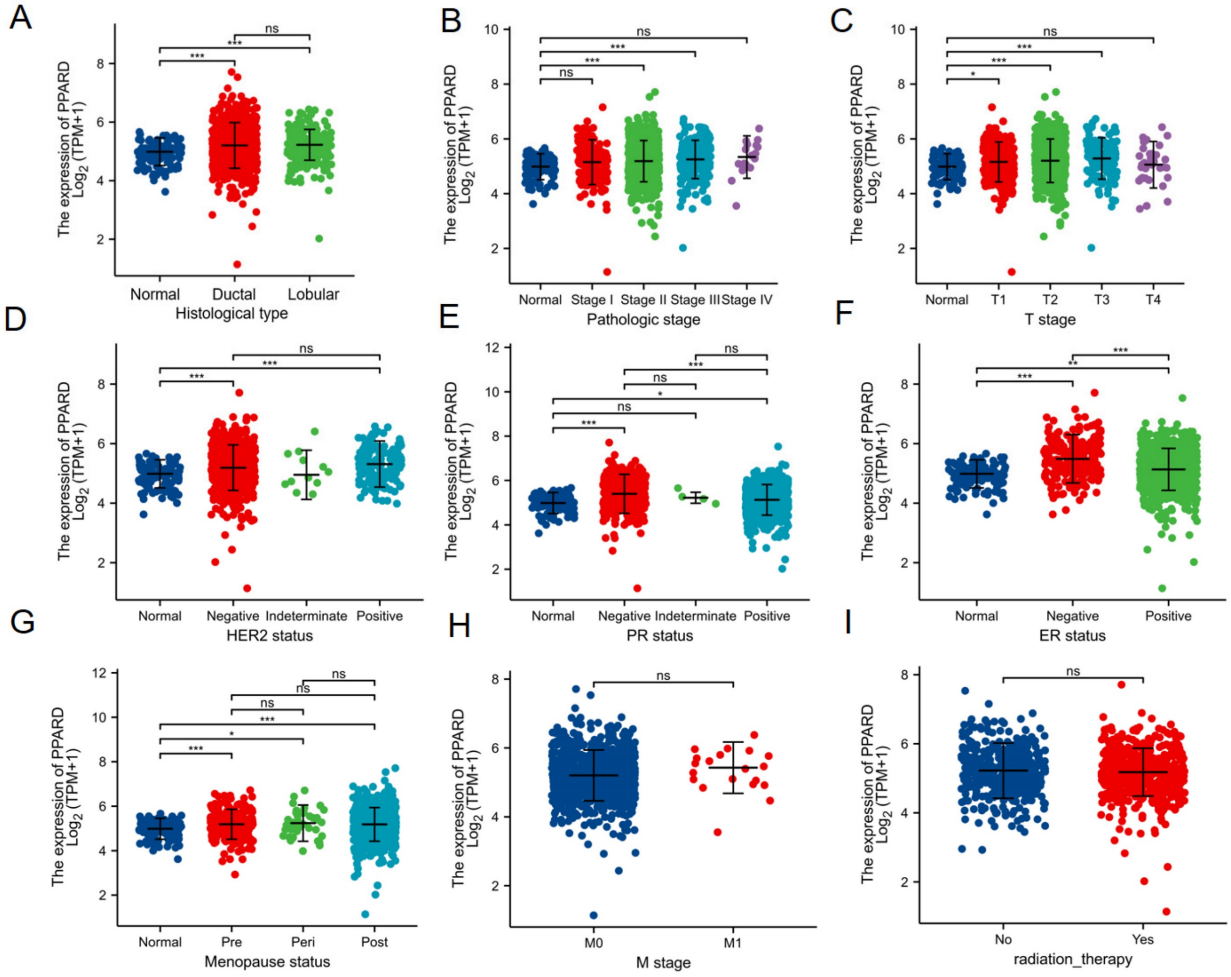
Association of PPAR-δ expression and clinicopathological characteristics. (**A**) histological type, (**B**) pathologic stage, (**C**) T stage, (**D**) HER2 status, (**E**) PR status, (**F**) PR status, (**G**) menopause status, (**H**) T stage and (**I**) radiation therapy.

To clarify the role and significance of PPAR-δ expression, a total of 1222 BRCA samples (TCGA database) with associated PPAR-δ expression and clinical data were analyzed. As shown in Figures 4A-E and Supplementary Table 1, PPAR-δ up-regulation is significantly correlated with tumor histological type (normal type vs. ductal type, P < 0.001; normal type vs. lobular type, P < 0.001), histological stage (normal vs. stage 2 and 3, P < 0.001), T stage (T1 vs. T2, T3, P < 0.05), Her2 stage (normal type vs. negative type, P < 0.001; normal type vs. positive type, P < 0.001), PR status (normal type vs. negative type, P < 0.001; normal type vs. positive type, P < 0.05), ER status (normal type vs. negative type, P < 0.001; normal type vs. positive type, P < 0.01; positive type vs. negative type, P < 0.001), menopause status (normal type vs. premenopausal, P < 0.001; normal type vs. perimenopausal, P < 0.05; normal type vs. postmenopausal, P < 0.001; perimenopausal type vs. postmenopausal, P > 0.05), M stage (M0 vs. M1 P > 0.05) and radiation therapy (none vs. completed, P > 0.05) (Figures 4E-I). These results suggest that BRCA with high PPAR-δ expression is more likely to progress to mid-to-late stage and is more sensitive to HER2 and ER mutations than BRCA with low PPAR-δ expression. Moreover, the results showed that PPAR-δ expression is closely related to a patient's physiological menopausal state. However, no correlation was found between PPAR-δ expression level and lymph node or distant organ metastasis, or between PPAR-δ expression and the use of radiotherapy in clinical treatment.

### High PPAR-δ expression is closely associated with a poor prognosis in BRCA patients

**Figure 5:**
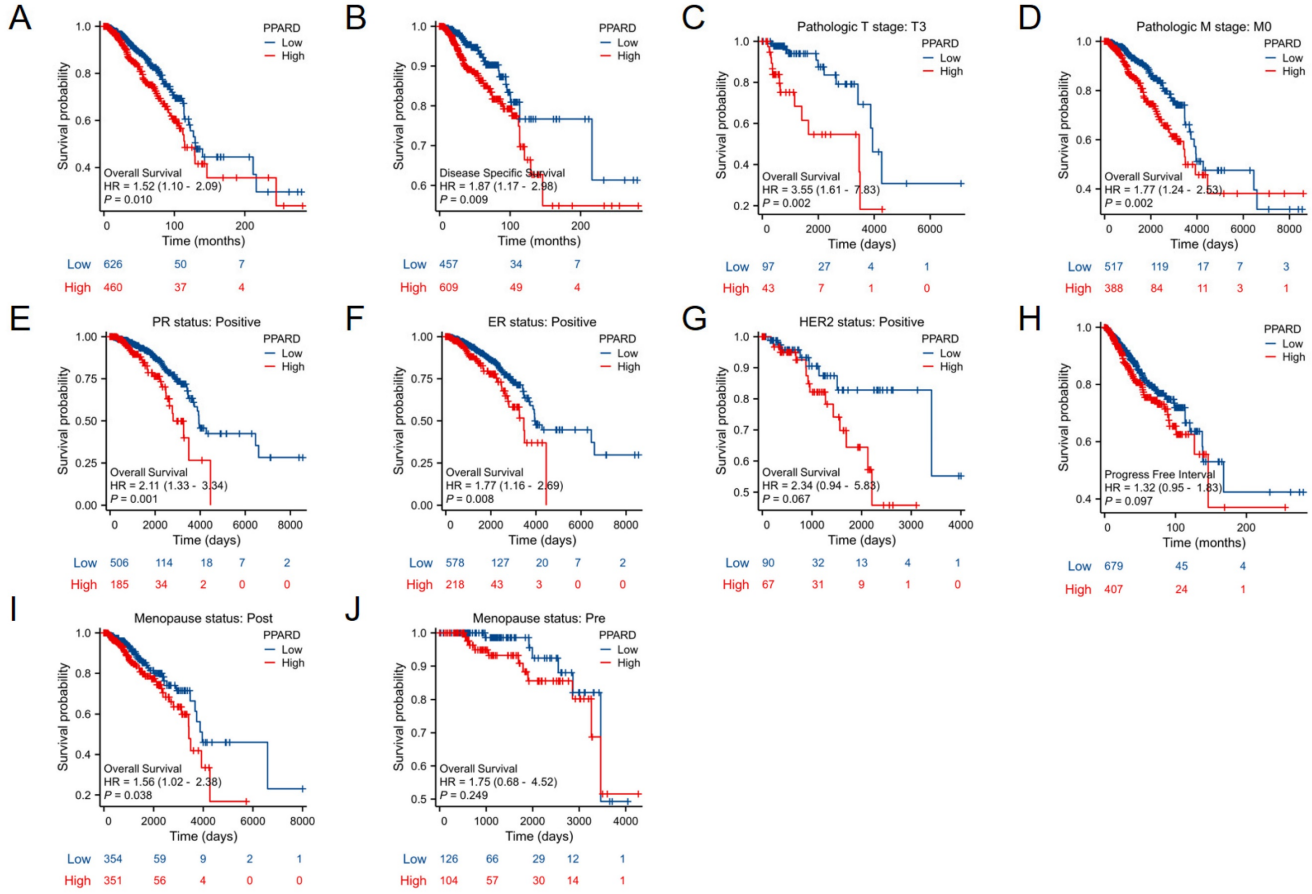
Kaplan-Meier survival curves comparing the PPAR-δ-low and PPAR-δ -high BRCA groups. Survival curves for (**A**) OS; (**B**) DSS; (**C**) T3; (**D**) M0; (**E**) PR stage positive; (**F**) ER stage positive; (**G**) Her stage positive; (**H**) PFI; (**I**) postmenopausal; (**J**) premenopausal OS, overall survival; DSS, disease specific survival; PFI, progression free interval.

When evaluating survival among the PPAR-δ expression groups, the 25-year OS rate was significantly higher in the low expression group relative to the high expression group (P = 0.014; Figure 5A). Similarly, the 25-year DSS was significantly higher in the low expression group when compared to the high expression group (P = 0.023; Figure 5B). Next, subgroup survival analysis of the OS was conducted and showed a poor patient prognosis for PPAR-δ-high patients that were in stage T3 or T4, PR positive, ER positive or HER2 positive. Furthermore, BRCA patients in the PPAR-δ-high group and in the M0 subgroup had a worse OS (P = 0.002), thus indicating that PPAR-δ has a greater prognostic role in BRCA patients without distant metastasis (Figures 5C-G). However, there was no significant difference in survival among the PFI (Figure 5H), menopause status (Figures 5I, J).

### Construction and validation of a PPAR-δ based nomogram

**Figure 6:**
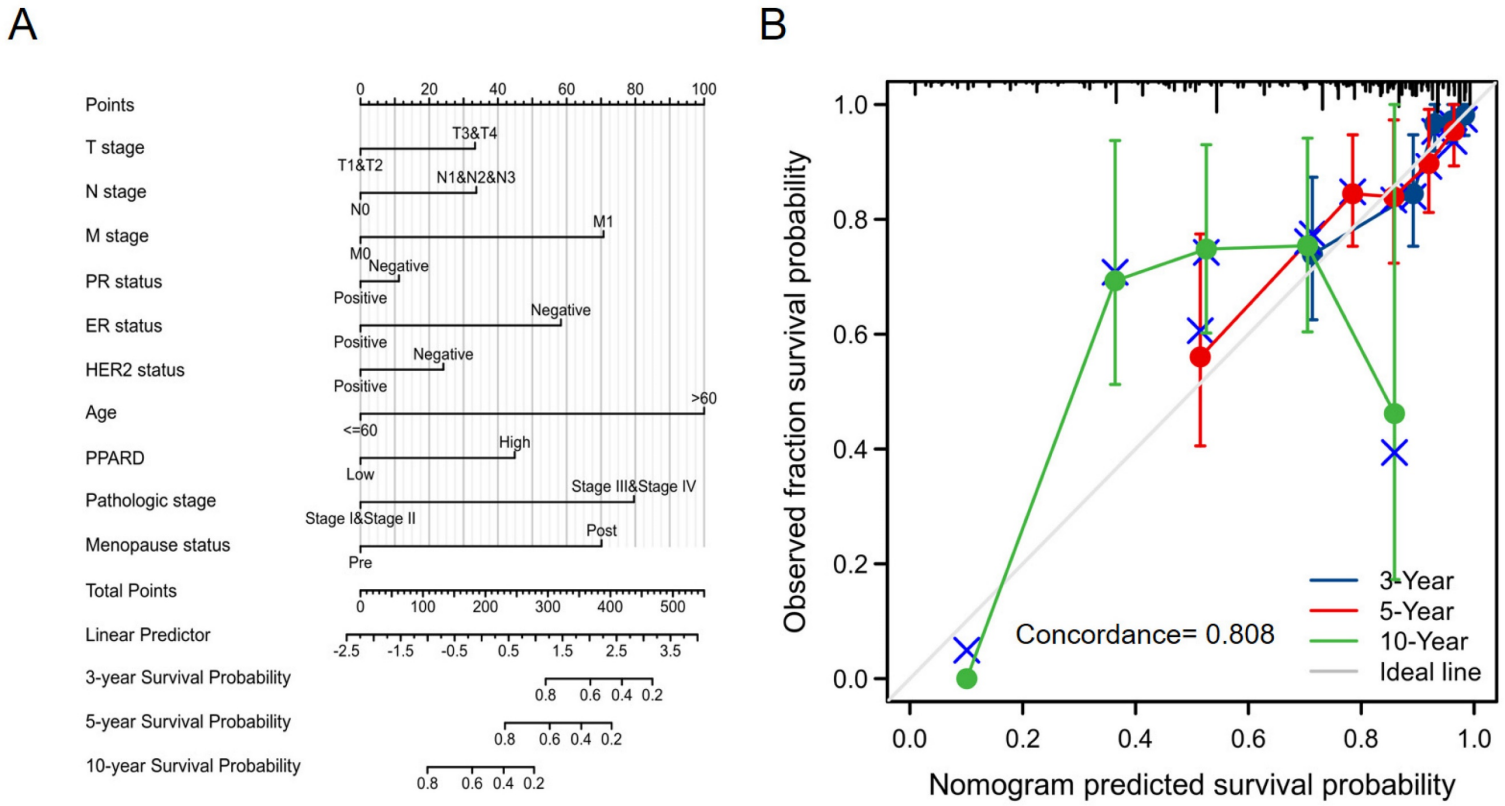
A quantitative method to predict BRCA patient OS probability at 3, 5, and 10 years. (**A**) A nomogram for predicting the probability of OS of 3, 5, and 10 years for BRCA patients. OS, overall survival. (**B**) Calibration plot based on the generated nomogram for predicting the probability of OS at 3, 5 and 10 years. The abscissa is the survival probability predicted by the model and the ordinate is the actual observed survival probability. Each point represents the model predicted survival probability and the actual observed survival probability. The gray diagonal line is the ideal line.

To provide a quantitative approach predicting BRCA patient prognosis, PPAR-δ and the independent clinical risk factors were used to construct a nomogram (Figure 6A). The nomogram was constructed based on a multivariate Cox regression analysis, with a point scale used and the sum of points assigned to each variable adjusted to a range from 1 to 100. The points from all of the variables were then tabulated to determine the total points. The probability of survival in BRCA patients at 3, 5 and 10 years was determined by drawing a vertical line directly down from the total points axis to the outcome axis. For instance, a BRCA patient with high PPAR-δ risk (45 points), ER risk (50 points), T risk (30 points), pathologic risk (80 points) and age risk (80 points) would receive a total point score of 285 and subsequent 3-, 5- and 10-year survival probabilities of ~ 80%, 60%, and < 20%. A C-index value of 0.808 was obtained, thus indicating an accurate and efficient prediction. Calibration curves for predicting 3, 5 and 10 years were constructed with a bias-corrected line (45° line) utilized and showed a fine agreement between the predicted and observed (bootstrap = 1,000 resampling, Figure 6B). These findings suggest that the Cox regression model is more accurate for predicting the 3- and 5-year survival probabilities, but less reliable for predicting the 10-year survival probability.

### PPAR-δ is associated with immune checkpoint alterations in BRCA

**Figure 7:**
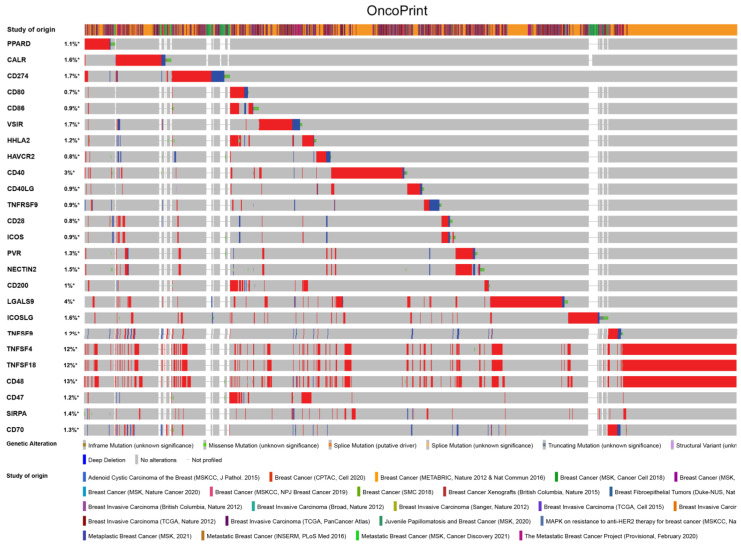
PPAR-δ couples with immune checkpoints in breast cancer. A condensed view of PPAR-δ and immune checkpoint alterations in BRCA. Genetic alterations are individually shown using cBioPortal, with fusion, amplification, deep deletion, truncating mutation, and missense mutations indicated. Brown represents inframe mutation. Green represents missense mutation. Orange represents splice mutation. Yellow represents splice mutation. Wathet represents truncating mutation. Purple represents structural variant. Blue represents deep deletion. And the longer the color bar, the greater the probability.

**Figure 8:**
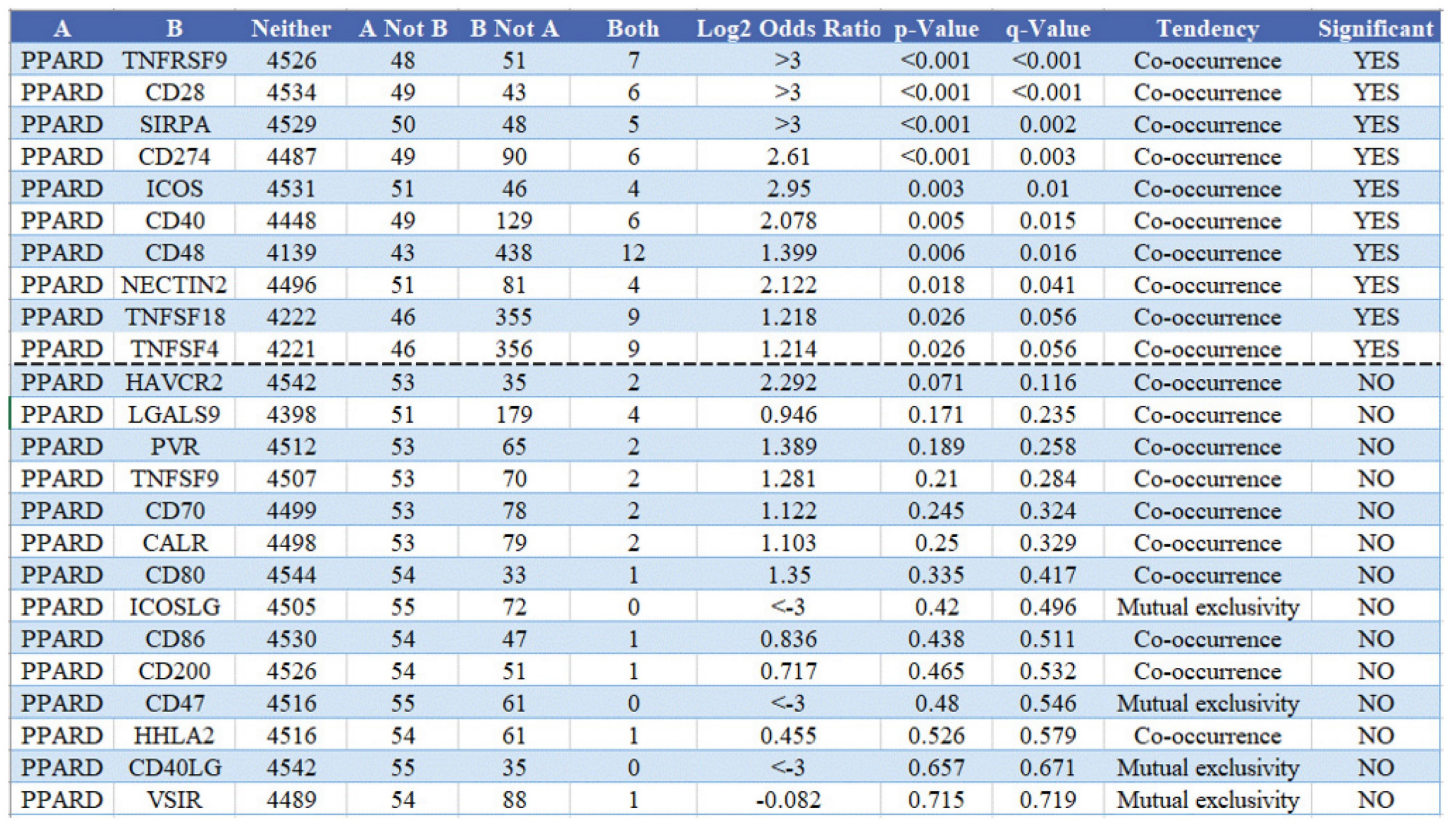
Mutual exclusivity analysis between PPAR-δ and multiple‐immune checkpoints in BRCA. The altered relationship between PPAR-δ and each immune checkpoint are indicated as a co‐occurrence or mutual exclusivity. The log_2_ odds ratio, P value, Q value, tendency, and significance are individually presented for each combination. Q and P values < 0.05 are considered statistically significant, with significance indicated with a “yes” and a lack of significance indicated with a “no.”

**Figure 9:**
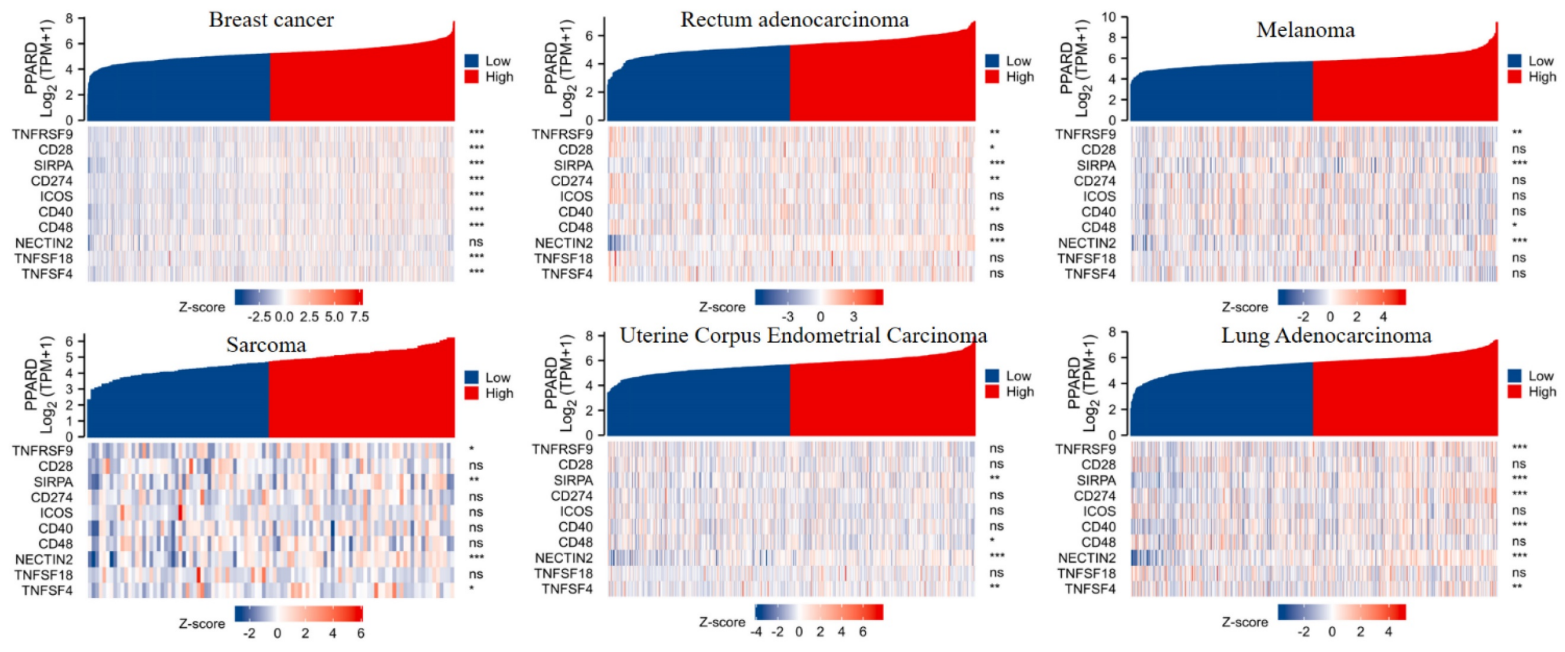
PPAR-δ and the immune checkpoints that were shown to have a significant co‐occurrence are also significant rectum adenocarcinoma, melanoma, sarcoma, uterine corpus endometrial carcinoma, lung adenocarcinoma and breast cancer.

To visualize an overview of genomic alterations in PPAR-δ and immune checkpoints in TCGA derived BRCA samples, OncoPrint 17 was employed. The results identified deep deletions, truncation mutations, missense mutations, in-frame mutations, splice mutations and structural variant. To establish potential correlations between PPAR-δ and each representative immune checkpoint, mutual exclusivity analysis was performed (Figure 7). Of note, the PPAR-δ alteration showed a statistically significant co‐occurrence rather than mutual exclusivity with a number of immune checkpoints, including TNFRSF9, CD28, SIRP-α, CD274, ICOS, CD40, CD48, TNFSF18, NECYIN and TNFSF4. These findings strongly suggest that PPAR-δ is a potential coregulator of immune checkpoints in BRCA (Figure 8). Moreover, the PPAR-δ alteration also shows a significant co‐occurrence with each of these same checkpoints when examining rectum adenocarcinoma, melanoma, sarcoma, uterine corpus endometrial carcinoma and lung adenocarcinoma samples (Figure 9). These similarities across various malignant tumors suggest a potentially broader PPAR-δ‐immune checkpoint interplay.

## Discussion

PPAR-δ is expressed in multiple tissues in the human body and plays a significant role in lipometabolism, inflammation, wound healing, keratinocyte differentiation and proliferation and cancer formation. While PPAR-δ expression has been shown to influence tumor growth, its potential as a prognostic indicator in BRCA has not been explored to our knowledge 18, 19. Hence, the aim of this study was to elucidate the role of PPAR-δ in BRCA and evaluate its potential as a prognostic indicator by utilizing bioinformatics analysis and *in vivo* and *in vitro* experimentation. Overall, the findings present herein indicate that PPAR-δ can serve as a prognostic survival prediction indicator in BRCA patients.

Interest in the potential impact of PPARs in cancer has been growing. The Primary Clinical Trials Database (https://clinicaltrials.gov) lists one clinical trial examining PPAR-α antagonists in various cancers and 24 clinical trials examining PPAR-γ modulators in cancer treatment, but none have examined PPAR-δ as a prognostic factor. Early experimentation examining the role of PPARδ activation in cancer growth were controversial, with one study showing that a PPARδ agonist (GW501516) enhances tumor growth in Apc(min) mice 20, while another study in the same journal and in the same year showed enhanced tumor growth in Apc(min) mice crossed with PPARβ/δ knockout mice 21. Numerous studies using different cell models have been published since then, with various aspects of PPAR-δ function and how it relates to cancer growth having been evaluated 18, 22-24.

Herein, PPAR-δ expression was found to be up-regulated in BRCA, with its overexpression significantly correlated with histological type, histological grade, Her2 stage, PR status and ER status. Moreover, BRCA patients with up-regulated PPAR-δ expression are more likely to progress to an intermediate or advanced stage and are more sensitive to HER2 and ER mutations than those with a lower PPAR-δ expression level. Additionally, while the PPAR-δ expression level was found to be closely associated with menopausal state, there was no association with lymph node or distant organ metastasis. These findings suggest that PPAR-δ is not closely related to breast cancer metastasis.

To further investigate the functions of PPAR-δ in BRCA, GO, KEGG, GSEA and ssGSEA analyses were performed using a BRCA TCGA data. The results revealed an association with protein digestion and absorption and estrogen signaling pathway, while PPAR-δ interactive genes were associated with antimicrobial humoral response, collagen catabolic processes and the transmembrane transporter complex. The differentially enriched pathways for the PPAR-δ-low expression group included PI3K/AKT, IL-18 and JAK/STAT pathways. After inhibiting PPAR-δ expression in a mouse model, the JAK/STAT signaling pathway was inhibited, with p-JAK significantly down-regulated in a dose-dependent manner. These data suggest that PPAR-δ might serve as a potential prognostic marker and therapeutic target in BRCA.

Immune cells and stromal cells in the tumor microenvironment can alter the oncogenic properties of tumor cells. Among them, tumor-infiltrating lymphocytes (TILs) play an important role in the occurrence and development of tumors. To enable tumor progression, a complex network of cell-to-cell interactions is established and promotes an immunosuppressive microenvironment and immune escape 25. Herein, CD56^bright^ NK cell levels were increased in the presence of lower PPAR-δ expression levels. CD56^bright^ NK cells express low levels of CD16A, which can produce a large amount of IFN-γ and other factors under the stimulation of cytokines to exhibit an anti-tumor effect 26. Thus, these findings suggest that inhibiting PPAR-δ expression can enhance the anti-tumor immune response via innate immune cells.

A high level of PPAR-δ expression was correlated with a poor prognosis when associated with stage T3 or T4, PR positive, ER positive or HER2 positive in BRCA patients. These results show that PPAR-δ expression can serve as a powerful prognostic predictor within these subsets, and that PPAR-δ acts independently of these important clinicopathological parameters. Furthermore, it should be noted that BRCA patients in the PPAR-δ-high group and M0 subgroup had a worse OS, thus indicating that PPAR-δ has a greater prognostic value in BRCA patients without distant metastasis.

To further evaluate potential correlations between PPAR-δ and other important clinical factors (PPAR-δ risk, ER risk, T risk, pathologic risk and age risk), a nomogram was constructed based on Cox regression analysis. The associated calibration plot showed a favorable consistency between the actual and predicted values for a 3-, 5- or 10-year OS. However, a more accurate predictive probability for 3- and 5-year OS, but the ability to predict a 10-year OS was not found to be as reliable. The model utilized herein is constructed based on the complementary perspective for respective tumors and provides a personalized score for individual patients. These findings indicated that this and future constructed nomograms can potentially be utilized as a valuable new prognostic method for clinicians.

Although these results improve our understanding of the relationship between PPAR-δ and BRCA, there are still some limitations. First, in order to fully elucidate the specific role of PPAR-δ in the development of BRCA, several clinical factors and parameters should be considered, such as the details of treatment. However, this information is lacking or inconsistent in public databases because experiments were conducted at different centers. Second, the number of healthy subjects used as controls was very different from the number of cancer patients in the current study, so additional studies are needed with a more balanced sampling scheme. Third, since the current study was only based on RNA sequencing data that was obtained from the TCGA database, it is necessary to further investigate the direct mechanism of PPAR-δ in BRCA cancer. Furthermore, while this study found that PPAR-δ can modulate the activation of JAK-STAT signaling, a more in-depth study into the mechanisms is still necessary.

While PPAR-δ has been shown to be expressed in several tissues within human body and is highly significant in the regulation of various functions, this study has provided further elucidation into the role of PPAR-δ and its potential role as a moderate BRCA predictive biomarker. Herein, to our knowledge, this is the first study that has shown that an up-regulation of PPAR-δ is significantly associated with BRCA progression, a poor OS and immune infiltration. These findings suggest that an up-regulation of PPAR-δ may promote tumorigenesis through abnormal inflammatory and immune responses. This further characterization of PPAR-δ in breast cancer is hoped to contribute to improved prognoses and aid in further elucidating the significance of the clinicopathological and molecular pathogenesis of breast cancer.

## Supplementary Material

Supplementary figures.

Supplementary table 1: TCGA breast cancer patient characteristics.

Supplementary table 2: 597 items of PPAR-δ related differential expressed Genes.

Supplementary table 3: Items of GO+KEGG enrichment analysis.

Supplementary table 4: Gene sets enriched in phenotype high.
