# Supplementary figures and images for: PPAR-δ as a prognostic biomarker and its association with immune infiltrates in breast cancer PPAR-δ as a prognostic biomarker and its association with immune infiltrates in breast cancer
Source: J Cancer. 2023 Apr 17;14(6):1049–61. doi: 10.7150/jca.81430 (PMC10158510; doi:10.7150/jca.81430)

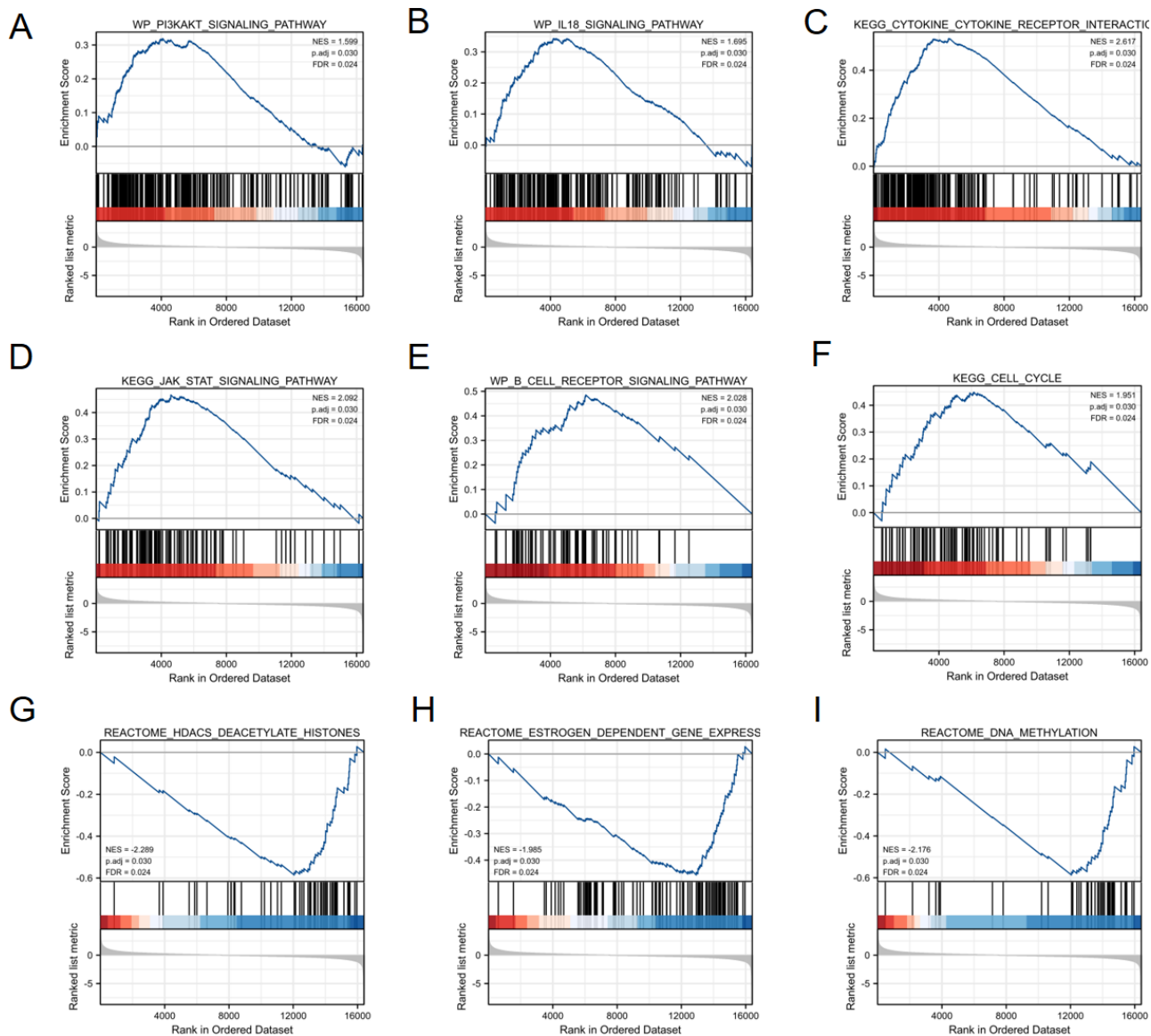

**SUPPLEMENTARY FIGURE 1**

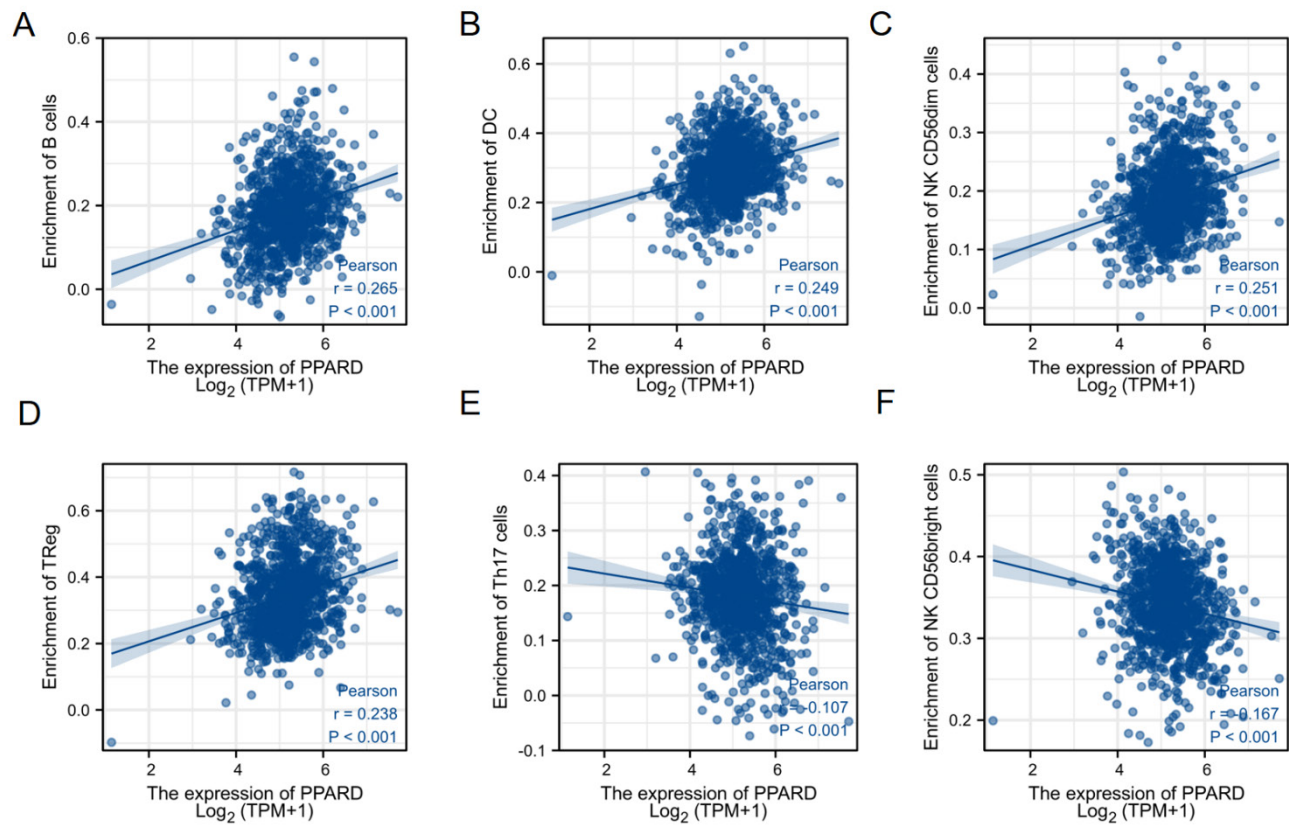

**SUPPLEMENTARY FIGURE 2**

Supplement: Supplementary file 1 — Supplementary figures. [file jcav14p1049s1.pdf]
