# Supplementary material for: PPAR-δ as a prognostic biomarker and its association with immune infiltrates in breast cancer PPAR-δ as a prognostic biomarker and its association with immune infiltrates in breast cancer
Source: J Cancer. 2023 Apr 17;14(6):1049–61. doi: 10.7150/jca.81430 (PMC10158510; doi:10.7150/jca.81430)
Supplement: Supplementary file 2 — Supplementary table 1: TCGA breast cancer patient characteristics. [file jcav14p1049s2.pdf]

Table 1. TCGA breast cancer patient characteristics.

Characteristic	Low PPAR-δ	High PPAR-δ	*P*
n	541	542	
T stage, n (%)			0.108
T1	147 (13.6%)	130 (12%)	
T2	310 (28.7%)	319 (29.5%)	
T3	60 (5.6%)	79 (7.3%)	
T4	22 (2%)	13 (1.2%)	
N stage, n (%)			0.038
N0	262 (24.6%)	252 (23.7%)	
N1	186 (17.5%)	172 (16.2%)	
N2	56 (5.3%)	60 (5.6%)	
N3	26 (2.4%)	50 (4.7%)	
M stage, n (%)			0.536
M0	447 (48.5%)	455 (49.3%)	
M1	8 (0.9%)	12 (1.3%)	
Pathologic stage, n (%)			0.404
Stage I	98 (9.2%)	83 (7.8%)	
Stage II	314 (29.6%)	305 (28.8%)	

Stage III	112 (10.6%)	130 (12.3%)	
Stage IV	8 (0.8%)	10 (0.9%)	
Race, n (%)			0.731
Asian	28 (2.8%)	32 (3.2%)	
Black or African American	86 (8.7%)	95 (9.6%)	
White	378 (38%)	375 (37.7%)	
Age, n (%)			0.120
<=60	287 (26.5%)	314 (29%)	
>60	254 (23.5%)	228 (21.1%)	
Histological type, n (%)			0.362
Infiltrating Ductal Carcinoma	384 (39.3%)	388 (39.7%)	
Infiltrating Lobular Carcinoma	94 (9.6%)	111 (11.4%)	
PR status, n (%)			< 0.001
Negative	126 (12.2%)	216 (20.9%)	
Indeterminate	2 (0.2%)	2 (0.2%)	
Positive	380 (36.8%)	308 (29.8%)	
ER status, n (%)			< 0.001
Negative	69 (6.7%)	171 (16.5%)	

Indeterminate	1 (0.1%)	1 (0.1%)	
Positive	438 (42.3%)	355 (34.3%)	
HER2 status, n (%)			0.065
Negative	280 (38.5%)	278 (38.2%)	
Indeterminate	7 (1%)	5 (0.7%)	
Positive	63 (8.7%)	94 (12.9%)	
PAM50, n (%)			< 0.001
Normal	12 (1.1%)	28 (2.6%)	
LumA	335 (30.9%)	227 (21%)	
LumB	114 (10.5%)	90 (8.3%)	
Her2	34 (3.1%)	48 (4.4%)	
Basal	46 (4.2%)	149 (13.8%)	
Menopause status, n (%)			0.931
Pre	116 (11.9%)	113 (11.6%)	
Peri	19 (2%)	21 (2.2%)	
Post	355 (36.5%)	348 (35.8%)	
Anatomic neoplasm subdivisions, n (%)			0.191
Left	270 (24.9%)	293 (27.1%)	
Right	271 (25%)	249 (23%)	

radiation_therapy, n (%)			0.430
No	211 (21.4%)	223 (22.6%)	
Yes	284 (28.8%)	269 (27.3%)	
OS event, n (%)			0.099
Alive	475 (43.9%)	456 (42.1%)	
Dead	66 (6.1%)	86 (7.9%)	
DSS event, n (%)			0.178
Alive	495 (46.6%)	483 (45.4%)	
Dead	36 (3.4%)	49 (4.6%)	
PFI event, n (%)			0.732
Alive	470 (43.4%)	466 (43%)	
Dead	71 (6.6%)	76 (7%)	
Age, median (IQR)	59 (49, 68)	57.5 (48, 66)	0.152
